# Synthesis, crystal structure and Hirshfeld surface analysis of *N*-(4-chloro­phen­yl)-5-cyclo­propyl-1-(4-meth­oxy­phen­yl)-1*H*-1,2,3-triazole-4-carboxamide

**DOI:** 10.1107/S2056989020005848

**Published:** 2020-04-30

**Authors:** Nazariy Pokhodylo, Yurii Slyvka, Volodymyr Pavlyuk

**Affiliations:** a Department of Organic Chemistry, Ivan Franko National University of Lviv, Kyryla i Mefodia Str, 6, 79005 L’viv, Ukraine; b Department of Inorganic Chemistry, Ivan Franko National University of Lviv, Kyryla i Mefodia Str, 6, 79005 L’viv, Ukraine

**Keywords:** crystal structure, 1,2,3-triazole, DFT calculation, Hirshfeld surface analysis

## Abstract

The title compound, C_19_H_17_ClN_4_O_2_, was obtained *via* a two-step synthesis involving the enol-mediated click Dimroth reaction of 4-azido­anisole with methyl 3-cyclo­propyl-3-oxo­propano­ate leading to the 5-cyclo­propyl-1-(4-meth­oxy­phen­yl)-1*H-*1,2,3-triazole-4-carb­oxy­lic acid and subsequent acid amidation with 4-chloro­aniline by 1,1′-carbonyl­diimidazole (CDI). It crystallizes in space group *P*2_1_/*n*, with one mol­ecule in the asymmetric unit. In the extended structure, two mol­ecules arranged in a near coplanar fashion relative to the triazole ring planes are inter­connected by N—H⋯N and C—H⋯N hydrogen bonds into a homodimer. The formation of dimers is a consequence of the above inter­action and the edge-to-face stacking of aromatic rings, which are turned by 58.0 (3)° relative to each other. The dimers are linked by C—H⋯O inter­actions into ribbons. DFT calculations demonstrate that the frontier mol­ecular orbitals are well separated in energy and the HOMO is largely localized on the 4-chloro­phenyl amide motif while the LUMO is associated with aryl­triazole grouping. A Hirshfeld surface analysis was performed to further analyse the inter­molecular inter­actions.

## Abstract 2

The title compound was obtained *via* a two-step synthesis involving the enole-mediated click Dimroth reaction of 4-azido­anisole with methyl 3-cyclo­propyl-3-oxo­propano­ate leading to the 5-cyclo­propyl-1-(4-meth­oxy­phen­yl)-1*H-*1,2,3-triazole-4-carb­oxy­lic acid and subsequent acid amidation with 4-chloro­aniline by 1,1′-carbonyl­diimidazole (CDI). In the extended structure, two mol­ecules arranged in a near coplanar position relative to the triazole ring planes are inter­connected by N—H⋯N and C—H⋯N hydrogen bonds into a homodimer. The dimers are linked by C—H⋯O inter­actions into ribbons.

## Chemical context   

The number of compounds containing a 1,2,3-triazolyl-4-carboxamide motif that are known to exhibit biological activity is increasing rapidly. At present, there are two approved drugs and a number of compounds are undergoing preclinical studies. For instance, rufinamide is a well-known drug among those currently marketed, which is used to treat Lennox–Gastaut syndrome (childhood-onset epilepsy) (Wheless & Vazquez, 2010 ▸). Carb­oxy­amido­triazole is a calcium channel blocker (Figg *et al.*, 1995 ▸) and is currently being actively investigated as an anti­cancer drug *in vitro* (Bonnefond *et al.*, 2018 ▸). As an example of preclinical anti­cancer studies, the cytotoxic activity at nanomolar levels of asymmetric 1-*R*-*N*-[(1-*R*-1*H-*1,2,3-triazol-4-yl)meth­yl]-1*H-*1,2,3-triazole-4-carb­oxamides in B16 melanoma cells have been estimated (Elamari *et al.*, 2013 ▸).

In our previous studies on the anti­cancer screening of various 1,2,3-triazoles, compounds based on 1,2,3-triazolyl-4-carboxamide scaffolds possessed the highest anti­proliferative activity (Shyyka *et al.*, 2019 ▸; Pokhodylo *et al.*, 2013 ▸, 2014 ▸). Furthermore, a series of 6,7-disubstituted-4-(2-fluoro­phen­oxy)quinoline derivatives possessing the 1,2,3-triazole-4-carboxamide moiety have been evaluated against *c*-Met kinase and five typical cancer cell lines (A549, H460, HT-29, MKN-45 and U87MG) and exhibited moderate to excellent anti­proliferative activity (Zhou *et al.*, 2014 ▸). A library of 1-benzyl-*N-*(2-(phenyl­amino)­pyridin-3-yl)-1*H-*1,2,3-triazole-4-carboxamides was screened for their anti­proliferative activity and showed promising cytotoxicity against lung cancer cell line A549 (Prasad *et al.*, 2019 ▸). In addition to the anti­tumor studies, 1*H-*1,2,3-triazole-4-carboxamides exhibit other biological activities such as fungicidal (Wang *et al.*, 2014 ▸), anti­viral (Krajczyk *et al.*, 2014 ▸) and anti­microbial (Jadhav *et al.*, 2017 ▸) activities and were found to be inhibitors of the Wnt/*β*-catenin signalling pathway (Obianom *et al.*, 2019 ▸). It should be noted that the diversity of such compounds can be obtained by amidation of 1*H-*1,2,3-triazole-4-carb­oxy­lic acids prepared by convenient Dimroth synthesis and further modifications (Pokhodylo *et al.*, 2009 ▸, 2017 ▸, 2018 ▸; Pokhodylo, Matiychuk *et al.*, 2010 ▸; Pokhodylo, Savka *et al.*, 2010 ▸; Pokhodylo & Obushak, 2019 ▸). Given the considerable inter­est in such scaffolds for drug discovery, a detailed study of their structural features is relevant and the crystal structure of the title compound, C_19_H_17_ClN_4_O_2_, is described herein.
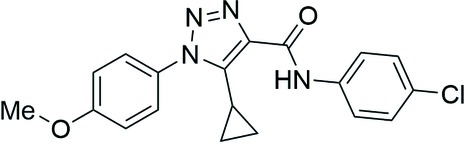


## Structural commentary   

**Figure 1:**
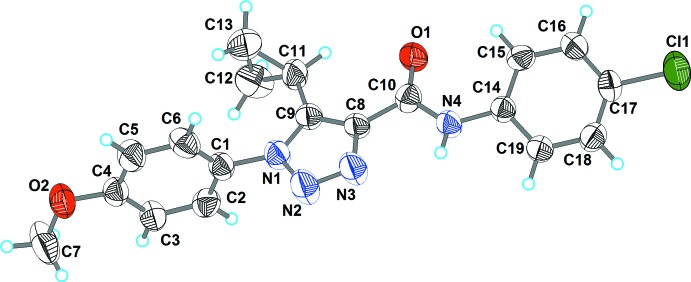
The mol­ecular structure of the title compound with displacement ellipsoids drawn at the 50% probability level.

The title compound crystallizes in the monoclinic centrosymmetric space group *P*2_1_/*n*, with one mol­ecule in the asymmetric unit. As shown in Fig. 1 ▸, the 4-meth­oxy­phenyl and 1,2,3-triazole rings are turned relative to each other by 87.77 (7)° because of a significant steric hindrance of the cyclo­propyl ring relative to the 4-meth­oxy­phenyl substituent [the N1—C9—C11—C13 and N1—C9—C11—C12 torsion angles are 41.2 (4) and −31.6 (4)°, respectively]. The above angle between the planes is comparable with that for the bulky 5-(2-phenyl­hydrazineyl­idene)methyl analogue [73.3 (2)°; Pokhodylo *et al.*, 2018 ▸] but is considerably larger than in the structure of 5-cyclo­propyl-1-(3-meth­oxy­phen­yl)-1*H-*1,2,3-triazole-4-carb­oxy­lic acid [39.1 (2)°] in which the cyclo­propyl ring is oriented to the triazole ring (Pokhodylo *et al.*, 2017 ▸) or in 5-methyl-1-(4-nitro­phen­yl)-1*H*-1,2,3-triazol-4-yl­phospho­n­ate [45.36 (6)°; Pokhodylo *et al.*, 2020 ▸]. In selected 5-free triazoles, 1-(3-bromo- or 4-fluoro­phen­yl)-1*H*-1,2,3-triazol-4-yl)methyl methyl­phospho­nates, this angle is 22.9 (3) and 15.7 (2)°, respectively (Pokhodylo, Shyyka *et al.*, 2019 ▸). Within the cyclo­propyl ring in the title compound, the three C—C bond lengths differ by an insignificant amount [C11—C12 = 1.491 (3), C11—C13 = 1.475 (3), C12—C13 = 1.457 (3) Å]. The amide group is turned slightly by 7.5 (3)° relative to the triazole ring while the proton of the amide group is involved in an intra­molecular hydrogen bond with the heterocyclic N3 atom (Table 1 ▸). The angle between the 4-chloro­phenyl and 1,2,3-triazole planes is 29.8 (1)°.

## Supra­molecular features   

**Figure 2:**
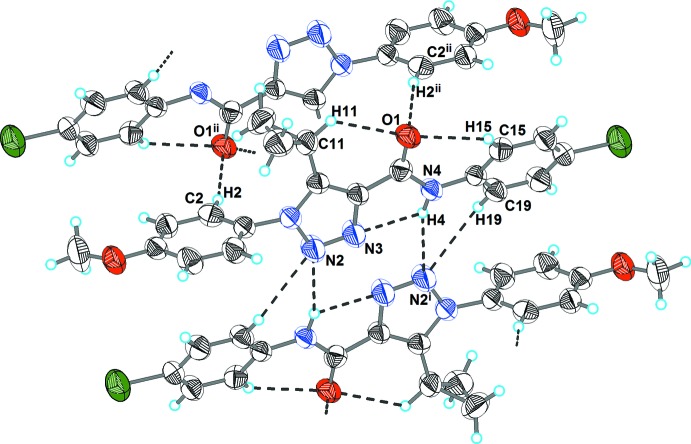
The hydrogen bonding of mol­ecules in the title compound. Hydrogen bonds are shown as dashed lines. The symmetry codes are as in Table 1 ▸.

**Figure 3:**
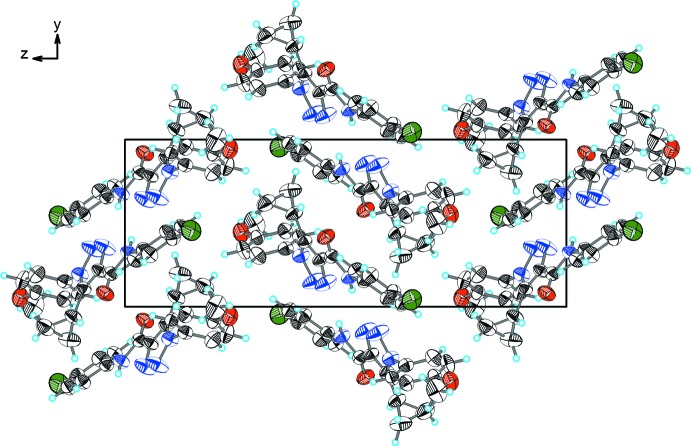
A view along the *a* axis of the crystal packing of the title compound.

As shown in Fig. 2 ▸ and Table 2 ▸, the extended structure of the title compound is consolidated by a number of inter­molecular inter­actions. Two mol­ecules arranged in a near coplanar manner relative to the triazole ring planes are inter­connected by N4—H4⋯N2^i^ and C19—H19⋯N2^i^ hydrogen bonds into a homodimer. Within the dimer, the edge-to-face stacked aromatic rings are tilted by 58.0 (3)°. Atom O1 of the amide group accepts both an intra­molecular C—H⋯O link (with the 4-chloro­phenyl and cyclo­propyl H atoms) and an inter­molecular C2—H2⋯O1 inter­action with the 4-meth­oxy­phenyl H atom. The last of these links neighbouring dimers into hydrogen-bonded ribbons parallel to the [010] direction (Fig. 3 ▸).

## Hirshfeld surface analysis and computational study   

**Figure 4:**
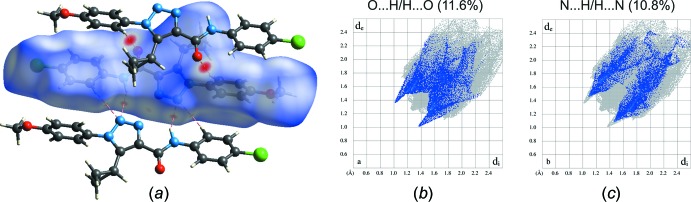
(*a*) Hirshfeld surface for the title mol­ecule mapped with *d*_norm_ over the range −0.171 to 1.473 a.u. showing N—H⋯N, C—H⋯N and C—H⋯O hydrogen-bonded contacts. Fingerprint plots resolved into (*b*) N⋯H/H⋯N and (*c*) O⋯H/H⋯O contacts. Neighbouring mol­ecules associated with close contacts are also shown.

Hirshfeld surface analysis was used to analyse the various inter­molecular inter­actions in the title compound, through mapping the normalized contact distance (*d*_norm_) using *CrystalExplorer* (Turner *et al.*, 2017 ▸; Spackman & Jayatilaka, 2009 ▸). Hirshfeld surfaces enable the visualization of inter­molecular inter­actions by using different colours and colour intensity to represent short or long contacts and indicate the relative strength of the inter­actions. The most prominent inter­actions (the *ortho*-proton of the aryl­triazole moiety and the carbonyl group as well as bifurcated inter­actions among protons of the amide group and the *ortho*-proton of the aryl group with the triazole ring nitro­gen (N2) atoms of neighbouring mol­ecules) can be seen in the Hirshfeld surface plot as red areas (Fig. 4 ▸). Fingerprint plots were produced to show the inter­molecular surface bond distances with the regions highlighted for (C)H⋯O and (C, N)H⋯N inter­actions (Fig. 4 ▸). The contribution to the surface area for such contacts are 11.6% and 10.8%, respectively.

**Figure 5:**
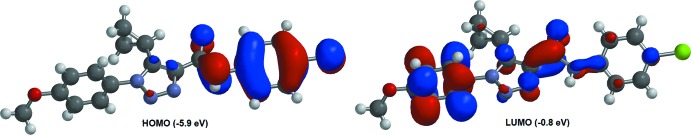
Frontier mol­ecular orbital energies.

The frontier mol­ecular orbitals HOMO and LUMO were analysed to better understand the electronic charge transfer within the mol­ecule and its electron donating and accepting ability. The mol­ecular orbital energies were calculated using the B3LYP functional level with the 6-31+G* basis set in a vacuum with *GAMESS* software (Schmidt *et al.*, 1993 ▸). The HOMO and LUMO orbitals were found to be well separated in energy and largely localized on the 4-chloro­phenyl amide or aryl­triazole motifs, respectively (Fig. 5 ▸). Their respective energy values were estimated to be −5.9 eV and −0.8 eV.

## Database survey   

The closest related compounds containing a similar 1-aryl-1*H-*1,2,3-triazole-4-carboxamide skeleton to the title compound but with different substituents on the amide are: (*S*)-1-(4-chloro­phen­yl)-*N-*(−1-hy­droxy-3-phenyl­propan-2-yl)-5-meth­yl-1*H-*1,2,3-triazole-4-carboxamide (I) (CCDC refcode: ZIPSEY; Shen *et al.*, 2013 ▸), 1-(4-chloro­phen­yl)-5-methyl-*N-*[(3-phenyl-1,2-oxazol-5-yl)meth­yl]-1*H-*1,2,3-triazole-4-carb­ox­amide (II) (LELHOB; Niu *et al.*, 2013 ▸), (5-methyl-1-(8-[tri­fluoro­meth­yl)quinolin-4-yl]-1*H-*1,2,3-triazol-4-yl)morph­o­lino)­methanone (III) (LOHWIP; Anuradha *et al.*, 2008 ▸) and 1-(3-amino-5-(3-hy­droxy-3-methyl­but-1-yn-1-yl)phen­yl)-*N-*butyl-1*H-*1,2,3-triazole-4-carboxamide (IV) (BEBJEZ; Li *et al.*, 2012 ▸).

Compounds (I) and (II) crystallize in the monoclinic crystal system [non-centrosymmetric space group *P*2_1_ in (I) and centrosymmetric *P*2_1_/c in (II)], while compounds (III) and (IV) crystallize in the triclinic space group *P* 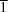. Structure (I) contains two crystallographically independent mol­ecules, the hydroxyl groups of which participate in inter­molecular O—H⋯O hydrogen bonds. In contrast to the structure of title compound, the dihedral angles between the phenyl rings and triazole rings in (I) are −45.2 (6)° (C5—C6—N1—N2) and 39.9 (6)° (C1′—C6′—N1′—N2′). The analogous angle in (II) is 19.2 (2)°. In structure (II), the carboxamide groups connect neighbouring mol­ecules into infinite hydrogen-bonded chains by means of N—H⋯O hydrogen bonds: these are linked by N—H⋯O (oxazole) contacts into a three-dimensional framework. Similarly to (I) and (II), structure (III) contains a 5-methyl substituent at the triazole ring and, because of significant steric hindrance of the 8-(tri­fluoro­meth­yl)quinoline group, the dihedral angle between the rings is 54.7°. The phenyl and triazole rings in (IV) are close to coplanar (7.5°), while the hydroxyl, carboxamide and amino groups participate in O—H⋯O and N—H⋯O hydrogen bonds. Finally, two copper(I) π-complexes with compositions [Cu(C_12_H_13_N_5_O)(NO_3_)]·0.5H_2_O and [Cu(C_12_H_13_N_5_O)(CF_3_COO)] (C_12_H_13_N_5_O is *N-*allyl-5-amino-1-phenyl-1*H-*1,2,3-triazole-4-carboxamide) were obtained by electrochemical synthesis (ZEQTOG and ZEQTUM; Slyvka *et al.*, 2012 ▸). Crystals of both compounds are monoclinic, space group *C*2/*c.* In both structures, the *N-*allyl-1*H-*1,2,3-triazole-4-carboxamide moiety acts as a bridging chelating ligand and forms, with the copper(I) atoms, infinite chains containing [CuC_4_NO] seven-membered rings.

## Synthesis and crystallization   

**Figure 6:**
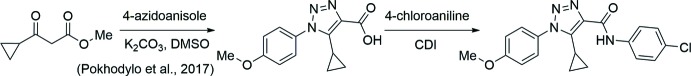
Synthesis of *N-*(4-chloro­phen­yl)-5-cyclo­propyl-1-(4-meth­oxy­phen­yl)-1*H-*1,2,3-triazole-4-carboxamide.

The title compound was synthesized from 5-cyclo­propyl-1-(4-meth­oxy­phen­yl)-1*H-*1,2,3-triazole-4-carb­oxy­lic acid (Pokhodylo *et al.*, 2017 ▸) by the following procedure (Fig. 6 ▸). 5-Cyclo­propyl-1-(4-meth­oxy­phen­yl)-1*H-*1,2,3-triazole-4-carb­oxy­lic acid **1** (1.3 g, 5.0 mmol) was added to a solution of 1,1′-carbonyl­diimidazole (0.81 g, 5.0 mmol) in dry aceto­nitrile (25 ml) and the mixture was kept for 30 min at 323 K. Then 4-chloro­aniline **2** (0.64 g, 5.0 mmol) was added, and the mixture was heated at 343 K for 1 h. After cooling to room temperature, water (30 ml) was added. The precipitate was filtered off, washed with water on a filter, recrystallized from ethanol solution, and dried in air to give the title compound as colourless prismatic crystals, m.p. 422–423 K; ^1^H NMR (500 MHz, DMSO-*d*_6_) δ 10.56 (*s*, 1H, NH), 7.89 (*d*, *J* = 8.6 Hz, 2H, H_Ar_), 7.58 (*d*, *J* = 8.6 Hz, 2H, H_Ar_), 7.39 (*d*, *J* = 8.6 Hz, 2H, H_Ar_), 7.16 (*d*, *J* = 8.6 Hz, 2H, H_Ar_), 3.86 (*s*, 3H, MeO), 2.10–1.99 (*m*, 1H, _cPr_CH), 0.95–0.80 (*m*, 4H, _cPr_CH_2_); ^13^C NMR (126 MHz, DMSO-*d*_6_) δ 160.62 (C=O or C_Ar_—O), 159.60 (C=O or C_Ar_—O), 142.26 (C_Triazole_-4), 138.87 (C_Triazole_-5), 138.21 (C^ClAr^-1), 129.08 (C_Ar_-1), 128.91 (2 × C_ClAr_-3,5), 127.77 (2 × C_Ar_-2,6), 127.70 (C_ClAr_-4), 122.25 (2 × C_ClAr_-2,6), 115.00 (2 × C_Ar_-3,5), 56.06 (MeO), 8.09 (2 × CH_2_^cPr^), 5.75 (CH^cPr^); MS *m/z* = 369 (*M* ^+^+1); Analysis calculated for C_19_H_17_ClN_4_O_2_ (*M*_r_ = 368.82), (%): C 61.88, H 4.65, N 15.19; found (%): C 61.91, H 4.74, N 15.21.

## Refinement   

Crystal data, data collection and structure refinement details are summarized in Table 2 ▸. All H atoms were positioned geometrically with N—H = 0.86 Å and C—H = 0.93–0.98 Å and refined as riding atoms. The constraint *U*_iso_(H) = 1.2*U*_eq_(carrier) or 1.5*U*_eq_(C-methyl carrier) was applied in all cases.

## Supplementary Material

Crystal structure: contains datablock(s) global, I. DOI: 10.1107/S2056989020005848/hb7901sup1.cif

Structure factors: contains datablock(s) I. DOI: 10.1107/S2056989020005848/hb7901Isup2.hkl

Supporting information file. DOI: 10.1107/S2056989020005848/hb7901Isup3.mol

Supporting information file. DOI: 10.1107/S2056989020005848/hb7901Isup4.cml

CCDC reference: 1999643

Additional supporting information:  crystallographic information; 3D view; checkCIF report

## Figures and Tables

**Table 1:** Hydrogen-bond geometry (Å, °)

*D*—H⋯*A*	*D*—H	H⋯*A*	*D*⋯*A*	*D*—H⋯*A*
N4—H4⋯N3	0.86	2.24	2.680 (3)	112
N4—H4⋯N2^i^	0.86	2.68	3.491 (2)	157
C15—H15⋯O1	0.93	2.39	2.936 (2)	117
C19—H19⋯N2^i^	0.93	2.68	3.475 (3)	144
C2—H2⋯O1^ii^	0.93	2.53	3.439 (3)	167
C11—H11⋯O1	0.98	2.47	3.124 (2)	124

Symmetry codes: (i) 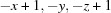; (ii) 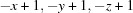.

**Table 2:** Experimental details

Crystal data	
Chemical formula	C_19_H_17_ClN_4_O_2_
*M*_r_	368.82
Crystal system, space group	Monoclinic, *P*2_1_/*n*
Temperature (K)	293
*a*, *b*, *c* (Å)	10.5673 (4), 8.0182 (3), 21.2318 (10)
β (°)	95.282 (4)
*V* (Å^3^)	1791.35 (13)
*Z*	4
Radiation type	Mo *K*α
μ (mm^−1^)	0.24
Crystal size (mm)	0.5 × 0.08 × 0.07

Data collection	
Diffractometer	Oxford Diffraction Xcalibur3 CCD
Absorption correction	Multi-scan (*CrysAlis RED*; Oxford Diffraction, 2005 ▸)
*T*_min_, *T*_max_	0.890, 0.982
No. of measured, independent and observed [*I* > 2σ(*I*)] reflections	10913, 3475, 1534
*R*_int_	0.046
(sin θ/λ)_max_ (Å^−1^)	0.617

Refinement	
*R*[*F* ^2^ > 2σ(*F* ^2^)], *wR*(*F* ^2^), *S*	0.040, 0.053, 1.05
No. of reflections	3475
No. of parameters	236
H-atom treatment	H-atom parameters constrained
Δρ_max_, Δρ_min_ (e Å^−3^)	0.14, −0.19

Computer programs: *CrysAlis PRO* (Oxford Diffraction, 2005 ▸), *SHELXT* (Sheldrick, 2015*a* ▸), *SHELXL* (Sheldrick, 2015*b* ▸) and *OLEX2* (Dolomanov *et al.*, 2009 ▸).

## supplementary crystallographic information

### Crystal data

**Table:**

C_19_H_17_ClN_4_O_2_	*F*(000) = 768
*M**_r_* = 368.82	*D*_x_ = 1.368 Mg m^−^^3^
Monoclinic, *P*2_1_/*n*	Mo *K*α radiation, λ = 0.71073 Å
*a* = 10.5673 (4) Å	Cell parameters from 1540 reflections
*b* = 8.0182 (3) Å	θ = 0.9–1.0°
*c* = 21.2318 (10) Å	µ = 0.24 mm^−^^1^
β = 95.282 (4)°	*T* = 293 K
*V* = 1791.35 (13) Å^3^	Prism, colourless
*Z* = 4	0.5 × 0.08 × 0.07 mm

### Data collection

**Table:**

Oxford Diffraction Xcalibur3 CCD diffractometer	1534 reflections with *I* > 2σ(*I*)
ω scans	*R*_int_ = 0.046
Absorption correction: multi-scan (CrysAlis RED; Oxford Diffraction, 2005)	θ_max_ = 26.0°, θ_min_ = 2.7°
*T*_min_ = 0.890, *T*_max_ = 0.982	*h* = −12→12
10913 measured reflections	*k* = −5→9
3475 independent reflections	*l* = −25→26

### Refinement

**Table:**

Refinement on *F*^2^	0 restraints
Least-squares matrix: full	Hydrogen site location: inferred from neighbouring sites
*R*[*F*^2^ > 2σ(*F*^2^)] = 0.040	H-atom parameters constrained
*wR*(*F*^2^) = 0.053	*w* = 1/[σ^2^(*F*_o_^2^) + (0.0071*P*)^2^ + 0.050*P*] where *P* = (*F*_o_^2^ + 2*F*_c_^2^)/3
*S* = 1.05	(Δ/σ)_max_ = 0.001
3475 reflections	Δρ_max_ = 0.14 e Å^−^^3^
236 parameters	Δρ_min_ = −0.19 e Å^−^^3^

### Special details

**Table:**

Geometry. All esds (except the esd in the dihedral angle between two l.s. planes) are estimated using the full covariance matrix. The cell esds are taken into account individually in the estimation of esds in distances, angles and torsion angles; correlations between esds in cell parameters are only used when they are defined by crystal symmetry. An approximate (isotropic) treatment of cell esds is used for estimating esds involving l.s. planes.

### Fractional atomic coordinates and isotropic or equivalent isotropic displacement parameters (Å^2^)

**Table:**

	*x*	*y*	*z*	*U*_iso_*/*U*_eq_	
Cl1	1.19945 (6)	0.04278 (8)	0.35044 (3)	0.0860 (2)	
O1	0.80102 (13)	0.41861 (18)	0.54511 (7)	0.0627 (5)	
O2	0.01792 (15)	0.44313 (19)	0.73741 (7)	0.0686 (5)	
N4	0.75389 (15)	0.1763 (2)	0.49150 (8)	0.0524 (5)	
H4	0.695819	0.101374	0.485272	0.063*	
N1	0.43143 (16)	0.3069 (2)	0.60195 (9)	0.0556 (5)	
C14	0.8626 (2)	0.1540 (2)	0.45845 (11)	0.0443 (6)	
C1	0.3243 (2)	0.3477 (2)	0.63661 (12)	0.0492 (6)	
C9	0.5403 (2)	0.3903 (2)	0.59331 (10)	0.0479 (6)	
C4	0.1154 (2)	0.4142 (3)	0.70082 (12)	0.0512 (6)	
C10	0.7294 (2)	0.3016 (3)	0.53206 (11)	0.0505 (6)	
N3	0.53626 (18)	0.1428 (2)	0.54590 (10)	0.0763 (7)	
C15	0.9796 (2)	0.2218 (2)	0.47738 (10)	0.0520 (6)	
H15	0.989197	0.290445	0.512804	0.062*	
C8	0.6050 (2)	0.2842 (3)	0.55768 (11)	0.0486 (6)	
C3	0.11310 (19)	0.4532 (2)	0.63777 (11)	0.0546 (6)	
H3	0.041147	0.501519	0.616770	0.066*	
N2	0.43009 (18)	0.1551 (2)	0.57229 (11)	0.0831 (7)	
C19	0.84942 (19)	0.0551 (3)	0.40452 (10)	0.0539 (6)	
H19	0.770296	0.011077	0.390662	0.065*	
C2	0.2188 (2)	0.4199 (3)	0.60555 (10)	0.0546 (6)	
H2	0.218048	0.446667	0.562890	0.066*	
C16	1.0831 (2)	0.1881 (3)	0.44386 (11)	0.0573 (7)	
H16	1.162198	0.233351	0.456901	0.069*	
C11	0.57821 (19)	0.5584 (3)	0.61661 (11)	0.0596 (6)	
H11	0.658079	0.596910	0.601492	0.072*	
C18	0.9525 (2)	0.0218 (2)	0.37150 (10)	0.0585 (7)	
H18	0.943421	−0.045418	0.335686	0.070*	
C17	1.0685 (2)	0.0881 (3)	0.39158 (11)	0.0533 (6)	
C5	0.2213 (2)	0.3398 (3)	0.73129 (11)	0.0635 (7)	
H5	0.222148	0.312257	0.773873	0.076*	
C6	0.3263 (2)	0.3056 (3)	0.69943 (12)	0.0623 (7)	
H6	0.397430	0.254642	0.720160	0.075*	
C13	0.5580 (2)	0.6216 (3)	0.68014 (12)	0.0782 (8)	
H13A	0.511849	0.550992	0.707204	0.094*	
H13B	0.625552	0.687084	0.702118	0.094*	
C12	0.4869 (2)	0.6965 (3)	0.62524 (13)	0.0809 (8)	
H12A	0.510672	0.808059	0.613084	0.097*	
H12B	0.396882	0.671863	0.618174	0.097*	
C7	−0.0993 (2)	0.4984 (3)	0.70643 (12)	0.0970 (9)	
H7A	−0.128382	0.419658	0.674241	0.146*	
H7B	−0.087613	0.605304	0.687433	0.146*	
H7C	−0.161144	0.507674	0.736641	0.146*	

### Atomic displacement parameters (Å^2^)

**Table:**

	*U*^11^	*U*^22^	*U*^33^	*U*^12^	*U*^13^	*U*^23^
Cl1	0.0723 (5)	0.0988 (5)	0.0925 (5)	0.0004 (4)	0.0379 (4)	−0.0087 (4)
O1	0.0573 (10)	0.0568 (10)	0.0765 (12)	−0.0187 (8)	0.0189 (9)	−0.0171 (9)
O2	0.0585 (11)	0.0879 (12)	0.0627 (12)	0.0026 (9)	0.0231 (10)	0.0082 (9)
N4	0.0475 (12)	0.0478 (12)	0.0639 (14)	−0.0110 (9)	0.0167 (11)	−0.0108 (10)
N1	0.0512 (13)	0.0499 (12)	0.0682 (15)	−0.0072 (11)	0.0186 (12)	−0.0094 (11)
C14	0.0454 (15)	0.0413 (14)	0.0468 (15)	−0.0044 (11)	0.0087 (14)	−0.0008 (12)
C1	0.0468 (16)	0.0458 (14)	0.0565 (18)	−0.0048 (12)	0.0130 (15)	−0.0036 (13)
C9	0.0480 (15)	0.0428 (14)	0.0534 (16)	−0.0059 (12)	0.0067 (14)	−0.0038 (12)
C4	0.0516 (17)	0.0527 (15)	0.0507 (17)	−0.0039 (12)	0.0127 (15)	0.0017 (13)
C10	0.0562 (17)	0.0463 (15)	0.0501 (16)	0.0002 (13)	0.0103 (15)	−0.0024 (13)
N3	0.0591 (14)	0.0633 (15)	0.1115 (19)	−0.0171 (11)	0.0347 (14)	−0.0363 (12)
C15	0.0512 (15)	0.0488 (15)	0.0553 (18)	−0.0033 (12)	0.0013 (15)	−0.0112 (12)
C8	0.0436 (15)	0.0443 (15)	0.0589 (17)	−0.0099 (12)	0.0110 (14)	−0.0123 (12)
C3	0.0493 (15)	0.0617 (15)	0.0540 (17)	0.0043 (12)	0.0109 (14)	0.0055 (13)
N2	0.0665 (16)	0.0618 (14)	0.127 (2)	−0.0215 (11)	0.0420 (15)	−0.0392 (13)
C19	0.0503 (15)	0.0561 (14)	0.0564 (16)	−0.0130 (12)	0.0109 (14)	−0.0093 (13)
C2	0.0619 (17)	0.0589 (15)	0.0434 (15)	−0.0041 (14)	0.0065 (15)	0.0055 (12)
C16	0.0456 (16)	0.0629 (16)	0.0644 (19)	−0.0073 (13)	0.0105 (15)	−0.0063 (14)
C11	0.0580 (16)	0.0533 (15)	0.0704 (18)	−0.0040 (13)	0.0210 (14)	−0.0185 (14)
C18	0.0657 (17)	0.0595 (16)	0.0520 (16)	−0.0095 (14)	0.0143 (15)	−0.0114 (12)
C17	0.0523 (16)	0.0561 (15)	0.0542 (17)	0.0004 (13)	0.0198 (14)	0.0034 (13)
C5	0.0602 (18)	0.0838 (18)	0.0474 (17)	−0.0001 (14)	0.0095 (16)	0.0168 (14)
C6	0.0478 (17)	0.0717 (17)	0.067 (2)	0.0024 (13)	0.0025 (16)	0.0143 (15)
C13	0.083 (2)	0.0669 (18)	0.085 (2)	−0.0152 (15)	0.0110 (19)	−0.0197 (16)
C12	0.073 (2)	0.0489 (16)	0.119 (2)	0.0045 (14)	−0.0003 (19)	−0.0099 (17)
C7	0.0580 (18)	0.136 (3)	0.102 (2)	0.0258 (17)	0.0314 (17)	0.0219 (19)

### Geometric parameters (Å, º)

**Table:**

Cl1—C17	1.742 (2)	C3—H3	0.9300
O1—C10	1.221 (2)	C3—C2	1.389 (3)
O2—C4	1.366 (2)	C19—H19	0.9300
O2—C7	1.419 (2)	C19—C18	1.375 (3)
N4—H4	0.8600	C2—H2	0.9300
N4—C14	1.412 (2)	C16—H16	0.9300
N4—C10	1.364 (2)	C16—C17	1.367 (3)
N1—C1	1.443 (2)	C11—H11	0.9800
N1—C9	1.358 (2)	C11—C13	1.475 (3)
N1—N2	1.370 (2)	C11—C12	1.491 (3)
C14—C15	1.376 (3)	C18—H18	0.9300
C14—C19	1.389 (2)	C18—C17	1.367 (3)
C1—C2	1.370 (3)	C5—H5	0.9300
C1—C6	1.374 (3)	C5—C6	1.379 (3)
C9—C8	1.363 (2)	C6—H6	0.9300
C9—C11	1.478 (3)	C13—H13A	0.9700
C4—C3	1.373 (3)	C13—H13B	0.9700
C4—C5	1.376 (3)	C13—C12	1.457 (3)
C10—C8	1.476 (3)	C12—H12A	0.9700
N3—C8	1.357 (2)	C12—H12B	0.9700
N3—N2	1.303 (2)	C7—H7A	0.9600
C15—H15	0.9300	C7—H7B	0.9600
C15—C16	1.385 (3)	C7—H7C	0.9600
			
C4—O2—C7	117.47 (18)	C15—C16—H16	120.1
C14—N4—H4	115.9	C17—C16—C15	119.8 (2)
C10—N4—H4	115.9	C17—C16—H16	120.1
C10—N4—C14	128.10 (18)	C9—C11—H11	113.1
C9—N1—C1	132.18 (19)	C9—C11—C12	124.1 (2)
C9—N1—N2	110.40 (17)	C13—C11—C9	124.2 (2)
N2—N1—C1	117.40 (17)	C13—C11—H11	113.1
C15—C14—N4	123.7 (2)	C13—C11—C12	58.84 (14)
C15—C14—C19	119.1 (2)	C12—C11—H11	113.1
C19—C14—N4	117.2 (2)	C19—C18—H18	120.2
C2—C1—N1	119.5 (2)	C17—C18—C19	119.7 (2)
C2—C1—C6	120.7 (2)	C17—C18—H18	120.2
C6—C1—N1	119.8 (2)	C16—C17—Cl1	119.63 (19)
N1—C9—C8	103.98 (17)	C16—C17—C18	120.8 (2)
N1—C9—C11	127.6 (2)	C18—C17—Cl1	119.56 (18)
C8—C9—C11	128.4 (2)	C4—C5—H5	119.6
O2—C4—C3	124.7 (2)	C4—C5—C6	120.8 (2)
O2—C4—C5	115.5 (2)	C6—C5—H5	119.6
C3—C4—C5	119.9 (2)	C1—C6—C5	119.1 (2)
O1—C10—N4	124.08 (19)	C1—C6—H6	120.5
O1—C10—C8	122.9 (2)	C5—C6—H6	120.5
N4—C10—C8	113.0 (2)	C11—C13—H13A	117.7
N2—N3—C8	108.94 (17)	C11—C13—H13B	117.7
C14—C15—H15	119.9	H13A—C13—H13B	114.8
C14—C15—C16	120.2 (2)	C12—C13—C11	61.14 (16)
C16—C15—H15	119.9	C12—C13—H13A	117.7
C9—C8—C10	130.9 (2)	C12—C13—H13B	117.7
N3—C8—C9	109.70 (18)	C11—C12—H12A	117.8
N3—C8—C10	119.4 (2)	C11—C12—H12B	117.8
C4—C3—H3	120.2	C13—C12—C11	60.02 (15)
C4—C3—C2	119.6 (2)	C13—C12—H12A	117.8
C2—C3—H3	120.2	C13—C12—H12B	117.8
N3—N2—N1	106.97 (17)	H12A—C12—H12B	114.9
C14—C19—H19	119.8	O2—C7—H7A	109.5
C18—C19—C14	120.4 (2)	O2—C7—H7B	109.5
C18—C19—H19	119.8	O2—C7—H7C	109.5
C1—C2—C3	120.0 (2)	H7A—C7—H7B	109.5
C1—C2—H2	120.0	H7A—C7—H7C	109.5
C3—C2—H2	120.0	H7B—C7—H7C	109.5
			
O1—C10—C8—C9	6.7 (4)	C4—C5—C6—C1	0.4 (3)
O1—C10—C8—N3	−173.6 (2)	C10—N4—C14—C15	23.3 (4)
O2—C4—C3—C2	179.10 (19)	C10—N4—C14—C19	−158.0 (2)
O2—C4—C5—C6	−179.5 (2)	C15—C14—C19—C18	1.7 (3)
N4—C14—C15—C16	177.1 (2)	C15—C16—C17—Cl1	−178.77 (17)
N4—C14—C19—C18	−177.10 (19)	C15—C16—C17—C18	0.6 (3)
N4—C10—C8—C9	−171.8 (2)	C8—C9—C11—C13	−139.8 (3)
N4—C10—C8—N3	7.9 (3)	C8—C9—C11—C12	147.4 (3)
N1—C1—C2—C3	177.23 (19)	C8—N3—N2—N1	−0.5 (3)
N1—C1—C6—C5	−177.6 (2)	C3—C4—C5—C6	0.8 (3)
N1—C9—C8—C10	179.6 (2)	N2—N1—C1—C2	−86.6 (2)
N1—C9—C8—N3	−0.1 (3)	N2—N1—C1—C6	89.8 (3)
N1—C9—C11—C13	41.2 (4)	N2—N1—C9—C8	−0.2 (2)
N1—C9—C11—C12	−31.6 (4)	N2—N1—C9—C11	179.1 (2)
C14—N4—C10—O1	1.2 (4)	N2—N3—C8—C9	0.4 (3)
C14—N4—C10—C8	179.7 (2)	N2—N3—C8—C10	−179.4 (2)
C14—C15—C16—C17	0.5 (3)	C19—C14—C15—C16	−1.6 (3)
C14—C19—C18—C17	−0.7 (3)	C19—C18—C17—Cl1	178.88 (17)
C1—N1—C9—C8	178.1 (2)	C19—C18—C17—C16	−0.5 (3)
C1—N1—C9—C11	−2.7 (4)	C2—C1—C6—C5	−1.2 (3)
C1—N1—N2—N3	−178.2 (2)	C11—C9—C8—C10	0.4 (4)
C9—N1—C1—C2	95.2 (3)	C11—C9—C8—N3	−179.3 (2)
C9—N1—C1—C6	−88.4 (3)	C5—C4—C3—C2	−1.2 (3)
C9—N1—N2—N3	0.4 (3)	C6—C1—C2—C3	0.8 (3)
C9—C11—C13—C12	−112.4 (3)	C7—O2—C4—C3	8.1 (3)
C9—C11—C12—C13	112.6 (3)	C7—O2—C4—C5	−171.63 (19)
C4—C3—C2—C1	0.4 (3)		

### Hydrogen-bond geometry (Å, º)

**Table:**

*D*—H···*A*	*D*—H	H···*A*	*D*···*A*	*D*—H···*A*
N4—H4···N3	0.86	2.24	2.680 (3)	112
N4—H4···N2^i^	0.86	2.68	3.491 (2)	157
C15—H15···O1	0.93	2.39	2.936 (2)	117
C19—H19···N2^i^	0.93	2.68	3.475 (3)	144
C2—H2···O1^ii^	0.93	2.53	3.439 (3)	167
C11—H11···O1	0.98	2.47	3.124 (2)	124

Symmetry codes: (i) −*x*+1, −*y*, −*z*+1; (ii) −*x*+1, −*y*+1, −*z*+1.
